# Exposure to Phosphates and Nitrites through Meat Products: Estimation of the Potential Risk to Pregnant Women

**DOI:** 10.3390/nu15122777

**Published:** 2023-06-16

**Authors:** Danijela Vranić, Jelena Milešević, Dejana Trbović, Mirjana Gurinović, Vladimir Korićanac, Milica Zeković, Zoran Petrović, Slavica Ranković, Dragan Milićević

**Affiliations:** 1 Institute of Meat Hygiene and Technology, Kaćanskog 13, 11040 Belgrade, Serbia; danijela.vranic@inmes.rs (D.V.); dejana.trbovic@inmes.rs (D.T.); vladimir.koricanac@inmes.rs (V.K.); zoran.petrovic@inmes.rs (Z.P.); 2 Centre of Research Excellence in Nutrition and Metabolism, Institute for Medical Research, National Institute of Republic of Serbia, University of Belgrade, Tadeuša Košćuška 1, 11000 Belgrade, Serbia; jelena.milesevic@gmail.com (J.M.); mirjana.gurinovic@gmail.com (M.G.); zekovicmilica@gmail.com (M.Z.); slavica.rankovic.imr@gmail.com (S.R.)

**Keywords:** food additives, nitrites, phosphates, pregnant women, meat products, exposure assessment

## Abstract

Diet during pregnancy is one of the most important nutritional challenges associated with some risks for the mother and the fetus. For the first time, the study aims to estimate long-term (2018–2022) exposure to nitrate and phosphates in Serbian pregnant women, based on individual consumption data and accurate values measured in frequently consumed meat products. For this purpose, seven types of meat products, consisting of 3047 and 1943 samples, were collected from retail markets across Serbia, to analyze nitrites and phosphorus content, respectively. These data were combined with meat product consumption data from the Serbian National Food Consumption Survey to assess dietary intake of nitrites and phosphate. The results were compared with the acceptable daily intake (ADI) proposed by the European Food Safety Authority. The average dietary exposure (EDI) to phosphorus ranged from 0.733 mg/kg bw/day (liver sausage and pate) to 2.441 mg/kg bw/day (finely minced cooked sausages). Considering nitrite intake, the major sources were bacon (0.030 mg/kg bw/day) and coarsely minced cooked sausages (0.0189 mg/kg bw/day). In our study, average nitrite and phosphorus exposure in the Serbian pregnant women population are far below the EFSA recommendations (ADI 0.07 mg/kg bw/day and 40 mg/kg bw/day, respectively).

## 1. Introduction

Even though meat and meat products are one of the most important contributors of the modern diet, it is well known that the nutritional profile of processed meat has been perceived as unhealthy due to the high levels of saturated fatty acids, cholesterol [1], or components that could be considered with negative health impacts (sulfites, nitrites, and sodium). Moreover, elevated consumption of processed meat and red meat has been associated with cardiovascular diseases, colorectal, stomach, prostate, and pancreatic cancer [2]. According to an epidemiological study, processed meat has been classified as carcinogenic to humans (Group 1) while red meat is probably carcinogenic to humans (Group 2A) [3]. Because of this, there is growing interested in the processed meat industry to reduce food additives that could be considered unhealthy [4].

Nitrates and nitrites (E249–E252) are food additives of concern for humans’ health because they may interact with secondary amines in the stomach, producing nitritesN-nitroso compounds (*N-NAs*), which could play a role in the carcinogenicity of processed meat [5,6]. Among meat products, cured meats often contain detectable levels of *N-NAs* mainly due to the use of nitrites as a preserving agent, additionally influenced by several processing factors (i.e., temperature, pH, and storage conditions) strongly linked by the presence of free amines, particularly biogenic amines [7]. 

Besides meat products, the occurrence of *N-NAs* has also been reported in other foods, such as processed vegetables, cereals, milk and dairy products, and alcoholic and non-alcoholic beverages, among others. Regarding other sources of *N-NAs* exposure, tobacco products (cigarettes, cigars) followed by products used in personal hygiene (cosmetics, hair products, lotions, shampoos, soaps, etc.) represent the important non-dietary exposure sources to *N-NAs* [6].

The high incidence of gastrointestinal cancer reported in the United Kingdom, Canada, Colombia, Chile, Japan, Denmark, and Italy has been correlated with elevated nitrite intake from food [8]. Moreover, nitrites and nitrates may cause methemoglobinemia, a blood disorder in which hemoglobin can carry oxygen but is unable to release it effectively to body tissues. It is also known that nitrites cross the placenta in pregnancy, causing methemoglobin formation in fetuses [9]. Some earlier studies have demonstrated the teratogenic effect of nitrites, emphasizing their toxicity and severe developmental defects on embryos or even spontaneous abortions [10]. An ADI is established for the additives that represent a concern for the consumers’ health. The European Commission, according to the Scientific Opinion of the Panel on Food Additives and Nutrient Sources added to Food (ANS) using a Benchmark Dose (BMD) approach, recommended an ADI of 0.07 mg nitrite ion/kg bw per day [7].

On the other hand, phosphates are used as food additives (E338–E341, E343, E450–E452) to improve food quality. Excessive intake of phosphates via consuming processed meat products can lead to various adverse effects on human health inducing anionic imbalance. An association between high serum phosphate levels and cardiovascular morbidity and mortality in patients with chronic kidney disease and bone health complications has long been known [11]. Therefore, high phosphorus intake from additives should be considered as a potential public health concern. For this purpose, the Scientific Committee for Food [11] derived a group acceptable daily intake (ADI) for phosphates expressed as phosphorus of 40 mg/kg bw/day and concluded that this ADI does not have adverse effects on human health.

Maximum permitted levels of food additives are set at the international level by the WHO-FAO JECFA and the European Food Safety Authority (EFSA) with the aim to ensure that additives are used properly to minimize potential risks to human health. Furthermore, under the European Directive [12], all Member States are obligated to monitor intakes to ensure that consumers do not have an excessive intake of a given food additive, which could lead to a health hazard. The current Serbian legislation has restricted the concentration of residual NaNO_2_ in processed meat to 100 and 150 mg/kg depending on the type of product [13,14], whereas regulations in Europe state that the maximum residual level (expressed as NaNO_2_) amount of nitrites that may be added to the processed meat during manufacturing should be from 50 to 180 mg/kg, particularly for dry-cured meat products such as bacon (175 mg/kg), for dry non-heat-treated meat products (50 mg/kg), and for other dry-cured meat products such as dry-cured ham (100 mg/kg), with several exemptions [12,15]. In terms of phosphorus used as a food additive, the Serbian standard maximum limit for total phosphorus expressed as P_2_O_5_ in meat products is less than 8 g/kg [14] or ≤5 g/kg of added phosphorus [13].

Diet during pregnancy is one of the most important nutritional challenges that may be associated with some risks for the health of mothers and the development of the fetus. In this context, a healthy and balanced diet is of the utmost importance during pregnancy and is an ongoing task for health care. Although nitrates and nitrites alone are considered to have no or limited carcinogenic potential [16], there are major human health concerns raised regarding nitrite intake, due to their potential conversion to form *N-NAs*. Based on the literature data, on associations between dietary intake of meat products, nitrite content, and cancer, the genotoxic properties of the *N-NAs* have been extensively investigated [6,17]. The high and frequent consumption of meat products, containing harmful substances such as nitrites, increases the risk of colorectal cancer and thyroid tumor promotion and adversely affects reproductive outcomes (e.g., fetal loss, reduced number of litters and live births, and neonatal mortality). Moreover, some studies reported a correlation between excessive dietary nitrite intake and a higher risk of development of neural tube defects [18,19] or even pediatric brain tumors in offspring [20].

Considering above mentioned rational and following our previous nitrites and phosphate studies [21,22,23], this study objective was to, for the first time, estimate dietary intake of nitrate and phosphates in Serbian pregnant women, based on individual consumption data and accurate values measured in most consumed groups of meat products. In addition, as a predictive model, i.e., “worse-case” scenario, values at the MPL was used in order to determine the level of reaching or exceeding ADI values for these two additives in meat products as a measure of identifying potential risk.

## 2. Materials and Methods

### 2.1. Meat Products and Sample Preparation

In the present study, 3047 meat product samples obtained from different regions of the Serbian retail market for the purposes of official controls by veterinary inspectors or for self-monitoring purposes of the meat producers during 2018–2022 were analyzed for nitrite content. Samples were divided into seven groups, including 381 bacon, 244 dry meat, 406 coarsely minced cooked sausages, 822 dry fermented sausages, 747 finely minced cooked sausages, 87 liver sausage and pate, and 423 smoked meat products, produced by the Serbian meat industry or imported. 

In the same period of investigation, a total of 1943 meat product samples were categorized into five groups including bacon (298), coarsely minced cooked sausages (405), finely minced cooked sausages (718), liver sausage and pate (86), and smoked meat products (436) were analyzed for phosphorous content. 

All samples of meat products were kept at refrigeration temperature and analyzed within 48 h. If the analyses were not conducted within the same day, the samples were stored in a refrigerator at 4 °C until required for testing. 

The analyzed samples were thawed and blended in a commercial kitchen blender unit (Homogenizator Blixer 2, Robot Coupe, Vincennes, France (2.9 L) 700 w, 3000 rpm). For each sample, two composite samples were prepared. All samples were then analyzed in duplicate.

### 2.2. Determination of Sodium Nitrite Content

The content of sodium nitrites-NaNO_2_, which is usually added to meat products—was examined in meat products according to the standard ISO procedure [24]. A representative sample amount (~10 g) was measured in a 300 mL flask using an analytical balance (Mettler, AE 200, Columbus, OH, USA), followed by the addition of a solution of hydrous sodium borate, Na_2_B_4_O_7_·10H_2_O (50 g/L) and 100 mL deionized water at 70.0 ± 0.2 °C. Residual nitrite extraction was achieved by keeping the samples in a hot water bath, at the temperature of boiling, for 15 min, and every 5 min, flasks were shaken vigorously. After cooling, 2 mL of each Carrez solution (Carezz reagent I and Carezz reagent II) was added and mixed thoroughly. Samples were then diluted to 200 mL with deionized water. Samples were filtered through quantitative cellulose filters (pore size < 5 μm). Color generation was achieved by transferring an aliquot of the filtrate (25 mL) to a 100 mL volumetric flask and adding 10 mL of the sulfanilamide solution and then 6 mL conc. HCl. Flasks were stored in the dark for 5 min. Subsequently, 2 mL solution of *N*-naftil-1-ethylenediamine-chloride (0.25 g/250 mL) was added to each flask and moved to the dark for 3 min. Thereafter, samples were diluted to 100 mL. Absorbance was measured at 538 nm using a spectrophotometer (UV/VIS Spectrophotometer, Jenway 6405, East Lyme, CT, USA). A procedural blank was run with every batch of samples.

Calibration curves were generated using concentration levels ranging from 2.5 to 10 NaNO_3_ μg mL^−1^, Y = 0.0669X + 0.024: R^2^ = 0.999. A recovery study of the analytical procedure was carried out by spiking several already analyzed samples with standard solutions, and recovery rates were found to be between 87% and 94%. The nitrite content is expressed as NaNO_2_ (mg·kg^−1^), following c × 2000/m × V, where c is the concentration of NaNO_2_ (μg/mL) from the calibration curve, m is the mass of sample (g) for analysis, and V is a volume of an aliquot of the filtrate used for spectrometric determination.

The limit of detection (LOD) was considered to take the same value as the limit of quantification (LOQ) (0.03 mg/kg).

### 2.3. Determination of Phosphorus Content

The total phosphorus content, expressed as P_2_O_5_ (g/kg), in examined meat products was determined according to the standard ISO procedure [25]. The total phosphorus content, expressed as P_2_O_5_ (g/kg), in examined meat products was determined according to the standard ISO procedure [25]. In brief, a ~5 g portion of samples (measured using an analytical balance (Mettler, AE 200, USA)) was ashed at the maximum temperature of 500 °C in a muffle furnace (LE 14/11/R7, Nabertherm, Lilienthal, Germany). On completion of the digestion, the white ash was dissolved by heating with dilute nitric acid (1 + 1, *v*/*v*) and quantitatively transferred to a 100 mL flask. Then, made up by the addition of deionized water, and after mixing, the solution was then filtered, and the first 5 to 10 mL were discarded. 

Aliquots (20 mL) of the treated solution were pipetted into 100 mL volumetric flasks and mixed thoroughly with 30 mL ammonium heptamolybdate solution 50 g/L. The resulting solution was then diluted to the volume with deionized water. After 15 min at room temperature, the absorbance was read against a reagent blank at 430 ± 2 nm using a UV-visible spectrophotometer (UV/VIS Spectrophotometer, Jenway 6405).

The standard curve was determined under the same conditions as those for the samples using potassium dihydrogen phosphate as a standard (10–60 P_2_O_5_ μg/mL; Y = 0.0187X − 0.0096: R^2^ = 0.9999). A recovery study of the analytical procedure was carried out by spiking several already analyzed samples with standard solutions, and recovery rates were found to be between 89% and 95%. The total phosphorus content is expressed as P_2_O_5_ (g/kg), following c/20 m, where c is the concentration of P_2_O_5_ (μg/mL) from the calibration curve and m is the mass of the sample (g) for analysis.

The LOD was estimated at 0.0024 g/kg, while the LOQ for phosphorus as P_2_O_5_ was 0.081 g/kg.

### 2.4. Meat Products Consumption Data

The National Food Consumption Survey on adults including pregnant women, in compliance with the EFSA EU Menu methodology [26], was conducted between 2017 and 2022 and included a total of 145 pregnant women. EFSA EU Menu methodology considers the use of set of questionnaires: a general questionnaire on sociodemographic and anthropometric characteristics of the participants, an age-appropriate Food Propensity Questionnaire (FPQ), that is used to determine the frequency of food groups’ consumption in a year, and a twice-repeated 24 h dietary recall. All the data are collected in the required format following the EU Menu framework, to provide harmonious and standardized data collection in all countries in Europe [27]. The consumed portion sizes were estimated based on natural units, household measures, packaging information, and a validated national Food Atlas for Portion Size Estimation [28]. The study was conducted in four geographical regions of Serbia (Belgrade, Vojvodina, Southeast Serbia, and West Serbia).

In this study, the following data were used: anthropometric characteristics of the participants, i.e., age, body weight, and height measurements, and intake data of meat products. This study assessed the consumption of meat products (per meat product type and on average) in a pregnant population. Consumed meat products were categorized into seven categories which were defined according to the actual Serbian Regulation on the quality of meat products [14]. The study group age is divided into two groups, to better describe characteristics of the population and age distribution, but is later not correlated in the exposure assessments, as both age groups belong to the same, adult population groups in the reference values—ADI and EDI—by the EFSA.

### 2.5. Exposure Assessment and Risk Characterization

According to the European Commission [29], there are three types of approaches to estimate the dietary exposure from food additives that pose a concern to human health:

(1) Tier 1: Estimation of the theoretical maximum daily intake by combining the maximum quantity of food and drinks that can be consumed by an individual with the maximum permitted level (MPL) of an additive.
(2) Tier 2: The use of individual consumption data multiplying with the MPL of the selected additives.
(3) Tier 3: The use of an individual Food Consumption Database (FCD) combined with the accurate measured values of selected additives.

The estimated daily intake (EDI) of nitrite and phosphate additives from processed meat by pregnant women included in this study was calculated using the Tier 3 approach, by combining data on individual food consumption patterns in pregnant women (g/day) with data on the levels of this type of additive in the investigated samples and division by the population’s average body weight (Table 1). Additionally, the mean value regarding the body weight of the investigated population of pregnant women obtained in our study is in accordance with EFSA recommendations, where a body weight of 70 kg should be used as the default for the European adult population [30].

In addition to the abovementioned method, the exposure assessment included certain assumptions of the worst-case scenario, so two levels of consumption were considered—mean and high consumer (P95 percentile)—assuming the maximum use level of these compounds defined by Serbian regulation [13,14] in meat processing combined with individual consumption data (Tier 2). For risk characterization, obtained results were then compared with the ADI values established by the European Union [7,11]. Relative contributions of processed meat products to the dietary intake of nitrites and phosphorus for pregnant women was expressed as a percentage of the ADI established at 0.07 mg/kg body weight/day and 40 mg/kg body weight/day, for nitrites and phosphorus, respectively. Taking into consideration adaptive changes in phosphorus metabolism that occur during pregnancy and lactation, the ADI for adults (40 mg/kg bw/day) could be also applied to pregnant and lactating women [31].

As international guidelines recommend [32] when calculating dietary exposure, all non-detected results, i.e., below the LOD or the LOQ, are known as left-censored. According to this guidance, for dietary exposure assessments where less than 60% of the results were left-censored, middle-bound (all non-detected results to the LOD/2) exposure scenarios were considered [32].

### 2.6. Statistical Analysis

Data were analyzed using Minitab statistical software version 17 (Minitab Ink., Coventry, UK). The results are presented in the form of descriptive statistics (mean ± standard deviation—SD) and their distribution (percentiles, and ranges). The normality of the distribution of the of the data were checked using by Kolmogorov–Smirnov normality test. One-way analysis of variance (ANOVA) followed by Tukey’s test was used to compare the dietary intake of phosphorous and nitrites among different meat products. The level of significance was set at *p* < 0.05.

## 3. Results

The mean and range of baseline characteristics of participants included in this study are presented in Table 1. The mean weight of the pregnant women included in this survey ranged from 70.34 ± 11.21 kg to 72.92 ± 13.93 kg (average 71.85 ± 12.90 kg). No statistically significant difference (*p* > 0,05) was observed between these two ages group of pregnant women in body weight. Regarding meat consumption, based on 145 participants interviewed, the highest average value of meat product consumption obtained in our research was for finely minced cooked sausages (84.83 ± 46.33 g/day), followed by dry fermented sausages (65.44 ± 64.22 g/day), while the lowest consumption was for bacon (30.84 ± 21.77 g/day). A statistically significant difference (*p* < 0.05) was found between the daily intake of bacon and finely minced cooked sausages and between the daily intake of finely minced cooked sausages and smoked meat products.

The mean, median, and 95th percentile levels of residual nitrites (NaNO_2_ and NO_2_^−^) and phosphorus (P_2_O_5_ and P^−^) in examined processed meat products over the period of 2018–2022 are summarized in Table 2 and Table 3. Nitrites were detected in 2443 (80%) of the total of 3047 analyzed meat product samples (Table 2). The results obtained in our study reveal that nitrite concentration varied with the type of meat product. The highest level of occurrence (99%) and mean residual level of nitrites, expressed as NaNO_2_, was detected in finely minced cooked sausages (38.72 ± 20.52 mg/kg), followed by coarsely minced cooked sausages (31.86 ± 23.30 mg/kg), while the lowest incidence (45%) and mean residual level of nitrite, as NaNO_2_, was detected in dry fermented sausages (1.44 ± 2.35 mg/kg). The average concentration of nitrites in the analyzed meat products was 19.56 ± 22.83 mg/kg. These results are far below the national Serbian or EU-regulated limit of 150 mg/kg [12,13]. In the current study, only one sample of smoked meat products exceeded the maximum permitted level of nitrites (data not shown). There were no statistically significant differences (*p* > 0.05) between the mean residual level of nitrite in bacon and liver sausage and pate, between dry meat and liver sausage and pate, and between dry meat and dry fermented sausages.

Phosphorus was detected in all analyzed meat product samples (1943) (Table 3). The average concentration of phosphorus, expressed as P_2_O_5_, in the analyzed meat products was 5.03 ± 1.37 g/kg within the range of 0.27 to 10.64 g/kg. The highest mean concentration and level of phosphorus, as P_2_O_5_, was found in smoked meat products (6.16 ± 1.38 g/kg and 10.64 g/kg, respectively), followed by coarsely minced cooked sausages (5.23 ± 1.14 g/kg and 9.92 g/kg, respectively), while the lowest mean concentration was found in liver sausage and pate (2.87 ± 0.95 g/kg). The results obtained in this study imply that the level of phosphorus in a total of 34 (1.7%) of the examined samples (except bacon and liver sausages and pate) exceeded the maximum permitted limit (MPL) of <8 g/kg as defined by the Serbian regulation [14] (Table 3).

**Figure 1:**
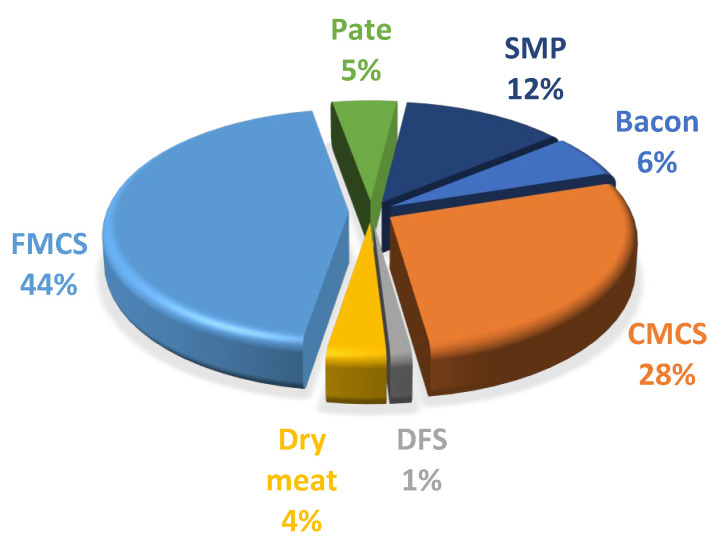
Relative contributions (%) of processed meat products to nitrite daily intake; CMCS—coarsely minced cooked sausages; DFS—dry fermented sausages; FMCS—finely minced cooked sausages; SMP—smoked meat products.

Exposure (mean, median, and 95th percentile) and the contribution of meat products to the daily nitrite intake of the pregnant women considered in this study are presented in Table 2 and Figure 1. Overall, dietary nitrite exposure at the mean and 95th percentile did not exceed the ADI for nitrite (0.07 mg/kg bw/day) [7] (Table 2). The main contributors to dietary exposure to nitrites were finely minced cooked sausages (43.54%), followed by coarsely minced cooked sausages (28%) and smoked meat products (12%), while the contribution of other groups was less than 10% (Figure 1).

**Figure 2:**
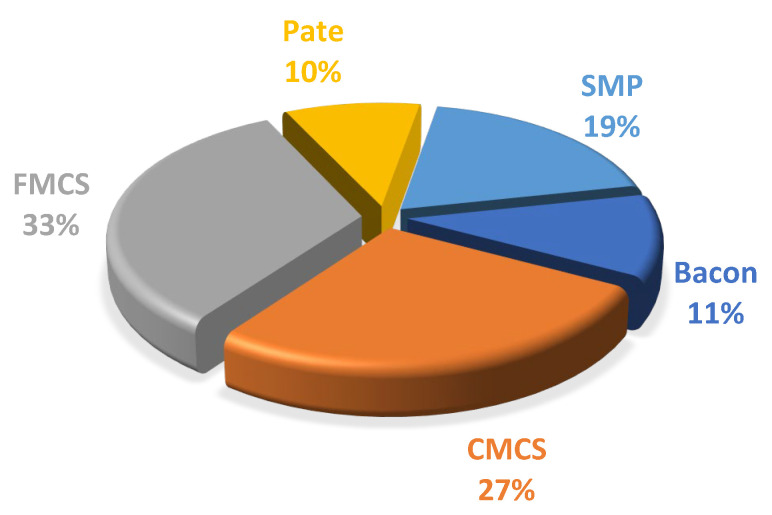
Relative contributions (%) of processed meat products to phosphorus daily intake; CMCS—coarsely minced cooked sausages; FMCS—finely minced cooked sausages; SMP—smoked meat products.

The main meat product contributing to dietary exposure to phosphates in our study was found to be finely minced cooked sausages, accounting for 33%, followed by coarsely minced cooked sausages (27%), smoked meat products (19%), bacon (11%), and liver sausage and pate (10%) (Figure 2). Hence, mean and 95th percentile exposure to phosphates in our study is far below this ADI (40 mg/kg bw/day) [11] (Table 3).

The results for the estimated daily intake and the relative percent contribution of each meat product included in this study to nitrite and phosphate exposure, combining individual consumption data with the MPL of the nitrite and phosphate additives (Tier 2 approach), are presented in Table 4 and Table 5. The major contributors to excess nitrite ADI are finely minced cooked sausages, followed by dry fermented sausages and coarsely minced cooked sausages, at 168.62%, 130.11%, and 127.25%, respectively (Table 4).

Concerning exposure to phosphates, in the worst-case scenario (Tier 2 approach), the meat products identified as the main contributor to phosphate intake were finely minced cooked sausages (10.30%), followed by coarsely minced cooked sausages (7.77%) and liver sausage and pate (5.10%) (Table 5). This is because these meat products were consumed in large quantities.

## 4. Discussion

The present study provides new information about pregnant women’s exposure to food-grade additives—nitrites and phosphorus via meat products. Pregnant women are considered more vulnerable to chemicals, particularly to ones which have carcinogenic and teratogenic properties because exposure occurs during the development of an embryo or fetus. Although nitrate and nitrites alone are considered to have no or limited carcinogenic potential [16], there are major human health concerns raised regarding nitrite intake, due to their potential conversion to form *N-NAs*. Based on the literature data, on associations between dietary intake of meat products, nitrite content, and cancer, the genotoxic properties of the *N-NAs* have been extensively investigated [6,17]. Although the primary sources of dietary nitrates and nitrites are vegetables, nitrates/nitrites from animal sources were attributable to an increase in cancer risk for the presence of amines, amides, and amides and heme iron that favor the increased production of *N-NAs* carcinogens. Consequently, there is a trend to reduce or eliminate these compounds in meats [33]. 

The present study showed a wide range of nitrite levels within and between the meat products at 0.05–180.25 mg/kg and is comparable to those reported by Nurul Farhanah Haji Abd Hamid [34] at 0.5–140.6 mg/kg. However, the mean and P95 residual nitrite levels in analyzed samples were below the maximum permitted limit specified by Serbian or EU regulations (150 mg/kg) [12,13]. These findings are consistent with previously reported nitrites content in sausages by Bajčić et al. [35] and Vranić et al. [22] at 0.65–36.60 mg/kg and 1.86–40.35 mg/kg (mean 12.96 mg/kg), respectively.

The dry fermented sausage samples were found to have the lowest amount of nitrites at 1.44 ± 2.35, followed by dry meat (4.86 ± 10.96 mg/kg), and these values were much lower compared to the other types of sausages. These findings are unexpected because the shorter shelf life was valid for coarsely or finely minced cooked sausage products, which were mostly less than 90 days, hence a lower amount of nitrate and nitrite additives were necessary to add. In this study, the highest health risks regarding nitrite intake by consuming meat products are in finely minced cooked sausages followed by coarsely minced cooked sausages (mean 0.0305 mg/kg bw/day and 0.0189 mg/kg bw/day, respectively). Consumption of finely minced cooked sausages at 152 g/day recorded in our study is of high concern, contributing to 0.054 mg/kg bw/day or 78.02% of the nitrite ADI (0.07 mg/kg bw/day), while the least risk was from dry fermented sausages with a level of 0.0009 mg/kg bw/day or 1.26% of the nitrite ADI. 

Phosphorus is an essential nutrient, occurring in foods of animal origin as a natural component and an approved ingredient added during food processing. Thus, JECFA proposed to assign a “maximum tolerable daily intake” (MTDI) rather than an ADI. The phosphorus EDI ranged from 0.733 to 2.445 mg/kg bw/day, representing 1.83 and 6.10% of the ADI specified by the EFSA [11]. The major contributor to phosphorus intake for pregnant women was finely minced cooked sausage (33% of phosphorus intake) and coarsely minced cooked sausage (27% of phosphorus intake) consumption. In both scenarios, the exposure does not exceed the ADI of 40 mg/kg bw per day (Table 3 and Table 5). However, ADI did not apply to populations with chronic kidney disease (CKD) or cardiovascular disease (CVD), considered a vulnerable population group. Thus, assessment of the EDI for those who consume phosphorus-rich food products regularly was important.

Although most authorized food additives are used at a lower level than the MPL, to ensure a high level of consumer protection, in addition we created a worst-case scenario for our risk assessment. The Tier 2 approach included certain assumptions of the worst-case scenario assuming the maximum use level of these food additives defined by the EU Regulation [12] in meat processing and the mean and highest percentile (95th percentile) of food-intake consumers. The Tier 2 intake estimates for nitrites and phosphorus are presented in Table 4 and Table 5. The differences between the results of nitrites and phosphates exposure obtained with two different exposure scenarios (Tier 2 and Tier 3 approaches) were significant. As expected, in the Tier 2 approach, exposure was considerably above the ADI. The major contributors to exceeding the ADI of nitrites and phosphates in this approach were finely minced cooked sausages, dry fermented sausages, and coarsely minced cooked sausages. These results could be explained by the fact that they were consumed in high quantities.

The strength of this study is in the fact that this exposure assessment determines a realistic dietary intake of nitrites and phosphorus additives based on data from national food consumption surveys and the concentrations of nitrites and phosphorus in each meat product measured analytically as practiced by EFSA [29]. From a broader perspective, these findings could be accepted as the most accurate reflection of current industry practices in Serbia. Furthermore, they complement and confirm the findings on nitrites and phosphorus content, obtained from laboratory analysis of meat products previously reported by the authors [21,22,23,35]. Bearing in mind that this provides new information about the dietary intake of nitrate and phosphates in Serbian pregnant women, using the method proposed by EFSA [29], the present survey has a lot of strengths and emphasizes the importance of monitoring the added amounts of food additives and why dietary exposure assessment must be continued. 

Considering the wide range of nitrites and phosphorus concentration levels within the meat products, we are aware of some limitations of our study. Thus, further study is necessary to consider the brand loyalty scenario. Very comprehensive studies revealed that consumers always tend to buy products of a given brand, which could have a higher concentration of additives than others [36]. Another limitation of this study lies in the circumstance that data on pregnancy state (trimesters) was not collected and correlation with exposure to observed additives could not be performed. This should be considered in the design of exposure studies in pregnancy in the future. 

The results of our study are not easily compared with others. To the best of our knowledge, so far, no studies on the exposure to nitrites and phosphorus by meat products in pregnant women were identified. In our study, the consumption of several meat product types exceeds the recommended intake (≤50 g/day) [37]. Consumption of industrially processed meat products, high in calories, fats, and salt with additives such as nitrites, has a cumulative detrimental effect on the overall health of pregnant women, i.e., an unnecessary increase in weight, swelling, water retention in the body, and can increase the risk of high blood pressure in pregnancy and the occurrence of preeclampsia. Balancing the diet with a wider variety of (less processed) foods could help consumers of this kind reduce their intake of nitrosamines.

## 5. Conclusions

Our study revealed that the population of pregnant women in Serbia is not at risk of exceeding the ADI for nitrites or phosphates from the consumption of processed meat. Furthermore, food-grade additive nitrites and phosphates as currently used in industry practices in Serbia do not result in excessive exposure to the populations of pregnant women, even at the highest food consumption level (95%). Despite this, these results should be interpreted with caution, as other dietary sources of nitrites and phosphorus must be considered. Results in our study confirm that the Tier 2 approach can lead to overestimated exposure to additives, because the measured level of nitrites and phosphates was far below the MPL in meat products. Although we used the representative National Food Consumption Database, it is reasonable to assume that eating habits tend to change over the years. Therefore, it is mandatory to establish monitoring systems for the use and intake of food additives to ensure that the ADI is not exceeded. The application of nitrites should be decreased and controlled. For this reason, a further investigation into the presence of *N-NAs* in food of animal origin will be of great interest. Besides this, the study demonstrates the need for community work on raising awareness and constant education on healthy nutrition during pregnancy that includes information on the detrimental effects that additives can have on infants and offers advice on alternative healthier dietary options. 

## Figures and Tables

**Table 1:** Baseline characteristics of study participants.

Age	*N*	Body Weight (kg)					
		P25	P50	P75	P95	Mean ± SD	Range (Min–Max)
18–29	60	62.0	70.0	75.7	89.9	70.3 ± 11.2 ^A^	50–107.1
30–43	85	64.2	71.0	78.0	97.6	72.9 ± 13.9 ^A^	50–141
Total	145	63.1	70.5	76.9	93.8	71.8 ± 12.9	50–141
Meat Product	Distribution of processed meat daily intake of the pregnant s population (g/day).						
Bacon		10.7	25.0	48.5	74.5	30.8 ± 21.7 ^A^	2.9–75.8
Dry meat		34.6	50.0	72.7	100.0	54.0 ± 27.8	12.8–125
Coarsely minced cooked sausages		35.0	50.0	100.0	100.0	64.0 ± 35.1	20–100
Dry fermented sausages		30.0	45.1	53.9	236.4	65.4 ± 64.2	25–240
Finely minced cooked sausages		46.8	72.5	137.5	152.0	84.8 ± 46.3 ^B^	36.6–152
Liver sausage and pate		28.8	42.5	50.0	72.5	42.0 ± 15.8	25–75
Smoked meat products		17.1	25.0	47.5	170.0	37.6 ± 39.7 ^A^	10–200
Average		22.45	41.69	55.02	150	49.58 ± 40.7	2.96–240

*N*—number of participants; Means with different superscripts in the same column are significantly different (*p* < 0.05).

**Table 2:** Ranges of residual nitrite levels expressed as NaNO_2_ and nitrite ion (NO_2_^−^), consumption of processed meat products (g/day), dietary exposure to nitrite (mg/kg bw/day), and relative contributions of processed meat products to nitrite exposure.

Meat Product	*N*	*n* (%)	Mean ± SD	P50	P95	Range (Min–Max)	ADC (g/day)	EDI (mg/kg bw/Day)		Contribution to ADI (%)	
			NaNO_2_(NO_2_^−^)(mg/kg)				Mean ± SD	Mean	P95	Mean	P95
Bacon	318	296 (93.1)	14.16 ± 17.72 ^A^ (9.44 ± 11.81)	6.61 (4.40)	53.20 (35.47)	<0.03–100.38 (<0.03–66.92)	30.84 ± 21.77 ^A^	0.0041	0.0094	5.79	13.45
Dry meat	244	168 (69.0)	4.86 ± 10.96 ^B,C^ (3.24 ± 7.31)	1.61 (1.07)	22.44 (14.96)	<0.03–97.30 (<0.03–64.87)	54.07 ± 27.88	0.0024	0.0056	3.49	7.99
Coarsely minced cooked sausages	406	396 (97.5)	31.86 ± 23.30 ^E^ (21.24 ± 15.53)	29.70 (19.80)	71.14 (47.43)	<0.03–113.51 (<0.03–75.67)	64.00 ± 35.07	0.0189	0.0296	27.03	42.23
Dry fermented sausages	822	372 (45.0)	1.44 ± 2.35 ^C^ (0.96 ± 1.57)	0.53 (0.35)	5.97 (3.98)	<0.03–24.88 (<0.03–16.59)	65.44 ± 64.22	0.0009	0.0032	1.26	4.54
Finely minced cooked sausages	747	742 (99.0)	38.72 ± 20.52 ^F^ (25.82 ± 13.68)	39.25 (26.17)	70.98 (47. 32)	<0.03–106.16 (<0.03–70.77)	84.83 ± 46.33 ^B^	0.0305	0.0546	43.54	78.02
Liver sausage and pate	87	69 (79.0)	8.49 ± 8.49 ^A,B^ (5.66 ± 5.55)	5.21 (3.47)	25.55 (17.03)	<0.03–30.39 (<0.03–20.26)	42.00± 15.85	0.0033	0.0053	4.73	7.60
Smoked meat products	423	400 (94.5)	23.94 ± 23.21 ^D^ (15.96 ± 15.47)	19.06 (12.71)	68.92 (45.95)	<0.03–180.25 (<0.03–120.17)	37.61 ± 39.76 ^A^	0.0084	0.0311	11.94	44.43
Average	3047	2443 (80.0)	19.56 ± 22.83 (13.04 ± 15.22)	7.62 (5.08)	63.36 (42.24)	<0.03–180.25 (<0.03–120.17)	49.58 ± 40.74	0.0098	0.0198	13.07	28.32

*N*—total number of analyzed samples; *n*-number of samples that contained nitrites (%); Nitrite ion content (66.65% of NaNO_2_); Means values with different superscripts in the same column are statistically significantly different (*p* < 0.05); ADC–average daily consumption of meat products (g/day); EDI—estimated daily intake (mg/kg bw/day); ADI—acceptable daily intake of nitrite ion (NO_2_^−^) (0.07 mg/kg bw/day) [7]; LOQ—limit of quantification = 0.03 mg/kg.

**Table 3:** Range of phosphorus levels (P_2_O_5_ and P), consumption of processed meat products (g/day), dietary exposure to phosphorus (mg/kg bw/day), and relative contributions of processed meat products to phosphorus exposure.

Meat Product	*N*	Mean ± SD	P50	P95	Range (Min–Max)	Above MPL (%)	ADC (g/day)	EDI (mg/kg bw/Day)		Contribution to MTDI (%)	
		P_2_O_5_ (P^−^), (g/kg)					Mean ± Sd	Mean	P95	Mean	P95
Bacon	298	4.40 ± 1.28 ^A^ (1.92 ± 0.48)	4.38 (1.91)	6.72 (2.93)	1.10–7.95 (0.48–3.47)		30.84 ± 21.77 ^A^	0.82	1.92	2.06	4.79
Coarsely minced cooked sausages	405	5.23 ± 1.14 ^B^ (2.28 ± 0.50)	5.12 (2.23)	7.31 (3.19)	2.25–9.92 (0.98–4.33)	7 (1.7)	64.00 ± 35.07 ^c^	2.04	3.18	5.09	7.95
Finely minced cooked sausages	718	4.74 ± 0.91 ^C^ (2.07 ± 0.40)	4.63 (2.02)	6.20 (2.71)	1.12–9.22 (0.49–4.02)	6 (0.8)	84.83 ± 46.33 ^B^	2.44	4.37	6.10	10.93
Liver sausage and pate	86	2.87 ± 0.95 ^D^ (1.25 ± 0.41)	2.77 (1.21)	4.08 (1.78)	0.27–7.96 (0.12–3.47)		42.00 ± 15.85	0.73	1.18	1.83	2.95
Smoked meat products	436	6.16 ± 1.38 ^E^ (2.69 ± 0.60)	6.12 (2.67)	7.98 (3.48)	1.01–10.64 (0.44–4.04)	21 (5.0)	37.61 ± 39.76 ^A^	1.41	5.24	3.52	13.09
Average	1943	5.03 ± 1.37 (2.19 ± 0.60)	4.88 (2.13)	7.67 (3.35)	0.27–10.64 (0.12–4.64)	34 (1.7)	45.94 ± 38.09	1.49	3.18	3.72	7.94

*N*—total number of analyzed samples; P content was 43.64% of P_2_O_5_; MPL—maximum permitted level (≤8 g/kg); Means with different superscripts in the same column are significantly different (*p* < 0.05); ADC—average daily consumption of meat products (g/day); EDI—estimated daily intake (mg/kg bw/day); MTDI—maximum tolerable daily intake of phosphorus (P) (40 mg/kg bw/day) [11].

**Table 4:** Scenario 2. Dietary exposure to nitrites by using actual national food consumption data and MPLs (Tier 2).

Meat Product	Daily Consumption of Meat Products (g/Day)			NaNO_2_ (NO_2_^−^) (mg/kg)	EDI (mg/kg bw/Day)			Contribution to ADI (%)		
	Mean ± SD	P50	P95		Mean	P50	P95	Mean	P50	P95
Bacon	30.84 ± 21.77	25.00	74.55	150 * (100)	0.043	0.035	0.100	61.32	49.71	142.48
Dry meat	54.07 ± 27.88	50.00	100.00		0.075	0.070	0.173	107.51	99.41	246.46
Coarsely minced cooked sausages	64.00 ± 35.07	50.00	100.00		0.059	0.070	0.139	127.25	99.41	198.83
Dry fermented sausages	65.44 ± 64.22	45.10	236.40		0.091	0.063	0.329	130.11	89.62	470.03
Finely minced cooked sausages	84.83 ± 46.33	72.50	152.00		0.118	0.101	0.212	168.67	144.15	302.22
Liver sausage and pate	42.00 ± 15.85	42.50	72.50		0.058	0.059	0.094	83.51	84.50	134.21
Smoked meat products	37.61 ± 39.76	25.00	170.00		0.052	0.035	0.195	74.78	49.70	278.36
Average	49.58 ± 40.74	41.69	150.00		0.075	0.101	0.177	107.59	88.07	253.23

* MPL—maximum permitted level of NaNO_2_ (150 mg/kg); EDI—estimated daily intake (mg/kg bw/day); ADI—acceptable daily intake of nitrite ion (NO_2_^−^) (0.07 mg/kg bw/day) [7].

**Table 5:** Scenario 2. Dietary exposure to phosphorus by using actual national food consumption data and MPLs (Tier 2).

Meat Product	Daily Consumption of Meat Products (g/Day)			P_2_O_5_ (P^−^) (g/kg)	EDI (mg/kg bw/Day)			Contribution to MTDI (%)		
	Mean ± SD	P50	P95		Mean	P50	P95	Mean	P50	P95
Bacon	30.84 ± 21.77	25.00	71.70	≤8 * (3.49)	1.500	1.215	3.482	3.75	3.04	8.70
Coarsely minced cooked sausages	64.00 ± 35.07	50.00	100.00		3.110	2.430	4.860	7.77	6.07	12.15
Finely minced cooked sausages	84.83 ± 46.33	72.50	152.00		4.122	3.523	7.390	10.30	8.81	18.46
Liver sausage and pate	42.00 ± 15.85	42.50	67.50		2.041	2.065	3.280	5.10	5.16	8.20
Smoked meat products	37.61 ± 39.76	25.00	140.00		1.830	1.215	6.803	4.57	3.04	17.01
Average	45.94 ± 38.09	36.50	150.70		2.520	2.089	5.162	6.30	5.22	12.90

* MPL—maximum permitted level for phosphorus (≤8 g/kg); MTDI—maximum tolerable daily intake of phosphorus (P) (40 mg/kg bw/day) [11].

## Data Availability

Results attained in this study are included in the manuscript. Individual data are not available due to official legal, organizational and data security policies, and ethical restrictions.
